# Collaborative project-based learning in global health: Enhancing competencies and skills for undergraduate nursing students

**DOI:** 10.1186/s12912-024-02111-8

**Published:** 2024-06-26

**Authors:** Sujin Lee, Ju Young Yoon, Yeji Hwang

**Affiliations:** 1 Department of Nursing, Kyungdong University, Wonju, Korea; 2 College of Nursing and Research Institute of Nursing Science, Seoul National University, 103 Daehak-ro, Jongno-gu, Seoul, 03080 Korea

**Keywords:** Global health, Nursing, Education, Project-based learning, Cooperation, Generative artificial intelligence

## Abstract

**Background:**

Despite the importance of collaboration and communication in global health, existing educational approaches often rely on traditional one-way instruction from instructor to student. Therefore, this study aimed to evaluate the effectiveness of a newly developed undergraduate curriculum on global health in enhancing nursing students’ competencies in global health and communication, problem-solving, and self-directed learning skills.

**Methods:**

A 15-week course “Global Health and Nursing” was designed for undergraduate nursing students, and a collaborative project-based learning method was used. Study participants were undergraduate nursing students enrolled in the course. The study was a multi-method study and included quantitative and qualitative components. It employed a one-group pretest–posttest design to quantitatively assess the impact of the curriculum. Additionally, student experiences with the learning process were qualitatively explored through a focus group interview. A total of 28 students participated in this study, and 5 of them participated in the focus group interview.

**Results:**

The collaborative project-based learning method significantly improved global health competency (*t* = − 10.646, *df* = 22, *p* < 0.001), with a large effect size. It also improved communication skills (*t* = − 2.649, *df* = 22, *p* = 0.015), problem-solving skills (*t* = − 3.453, *df* = 22, *p* = 0.002), and self-directed learning skills (*t* = − 2.375, *df* = 22, *p* = 0.027). Three themes were found through the focus group interview: (a) Promoting global health competency; (b) Fostering life skills through collaborative projects; and (c) Recommendations for future classes. The focus group interview indicated that overall, the study participants were satisfied with the collaborative project-based method for global health education.

**Conclusions:**

This study confirms that project-based learning significantly boosts the competencies and skills of students, recommending its broader adoption in nursing education. Nursing instructors should consider adopting this teaching approach for global health education at the undergraduate level. Future studies may employ a longitudinal design to assess the prolonged effects of the collaborative project-based learning approach, particularly focusing on the long-term retention of skills and the broader applicability of this model across different educational settings.

## Introduction

Global health problems, such as infectious diseases and intensifying health inequality, have arisen due to the acceleration of globalization. Thus, it has been recommended that prospective medical personnel should obtain interest and globalized competencies in areas such as disease characteristics, epidemiology, and health polarization across countries [1, 2]. The global health-related curriculum has been continuously developed in response to the demands of each era [3–5]. Starting with the International Federation of Medical Students’ Associations established in 1951, student participation and discussions to improve global health are actively taking place in various European countries [6]. Since the mid-1990s, global health education courses have been developed in countries such as Sweden, the United Kingdom, and the Netherlands [6]. In North America, after the establishment of the Global Health Education Consortium for International Health Education in 1991, the Consortium of Universities for Global Health was formed in 2008, and various academic systems and related organizations have cooperated to share information and develop global health education [7].

Project-based learning is a learner-centered teaching method in which the learner actively learns through autonomous goal setting, collaboration, communication, and reflection on practical cases [8, 9]. Project-based learning is characterized by viewing students as active subjects in the learning process and respecting their knowledge, perspectives, and experiences [10]. In health-care education, project-based learning has been considered a remarkable method for training health-care personnel [11, 12]. In this method, individuals and teams work collaboratively to improve the completeness of results through continuous team communication; students discover and develop their strengths in the process of understanding and solving project problems [13, 14]. These characteristics can have a meaningful effect on the development of health-care personnel’s capabilities to collaborate with experts in various fields in the rapidly changing global health field [15].

According to previous literature on global health competencies, common features include collaboration, partnering, communication, self-directed and ongoing learning, and identifying innovative solutions to global health problems [16, 17]. Therefore, when teaching global health, nurse educators need to consider improving the students’ global health competencies, communication, self-directed learning, and problem-solving skills so that they can become experts with sufficient competence in the global community. Problem-oriented learning is often suggested for global health education [17, 18]. However, most global health education continues to use the one-way traditional teaching method from instructor to student [19], which is more limiting than the global health experience that can be attained through collaboration [2]. Traditional lecture-based teaching methods often restrict the development of critical thinking or problem-solving skills, both of which are essential components in the field of global health [20]. While it is crucial to educate nurses to effectively practice in various areas of the global community, the existing literature provides limited evidence on the impact of project-based learning in enhancing global health competencies among nursing students.

Therefore, the overarching aim of this study was to evaluate the effectiveness of a newly developed undergraduate nursing course in global health; the course used collaborative project-based learning methodology. Specifically, the study aims were: (1) to quantitatively evaluate the change in global health competency, communication skills, problem-solving skills, and self-directed learning skills before and after the implementation of the course; and (2) to qualitatively investigate the undergraduate nursing students’ overall experiences with the course. For the quantitative components, we hypothesized that the collaborative project-based learning method would improve global health competency, communication skills, problem-solving skills, and self-directed learning skills.

## Methods

### Study design

This study was a multi-method study and included quantitative and qualitative approaches [21]. It used a one-group pretest–posttest design to examine the effectiveness of a global health course that employed collaborative project-based learning. Additionally, a focus group interview was conducted after the course ended to explore the undergraduate nursing students’ overall experience with the course.

### Study participants

The participants of this study were undergraduate nursing students from a university located in Seoul, Korea, enrolled in the course “Global Health and Nursing” during the fall semester of 2023. A total of 45 students enrolled in the course were approached for participation in this study. At the beginning of the study, its purpose and procedures were outlined to the students by the first author of this study (SL), who was not teaching the course. To ensure that participation was free of any undue pressure, the course director (YH) was not present during the information session.

The students were informed that participation was voluntary and that there would be no negative consequences to them if they did not participate in this study. Students who were 18 years old or older, enrolled in the course, and willing to participate were included in the study. Students who failed to complete the course (e.g., having more than five absences, not submitting assignments, or dropping out of the course) were excluded from the study. Consents to participate in the study were obtained from all participants, ensuring they were aware of their rights and the confidentiality measures taken to protect their personal information.

**Fig. 1:**
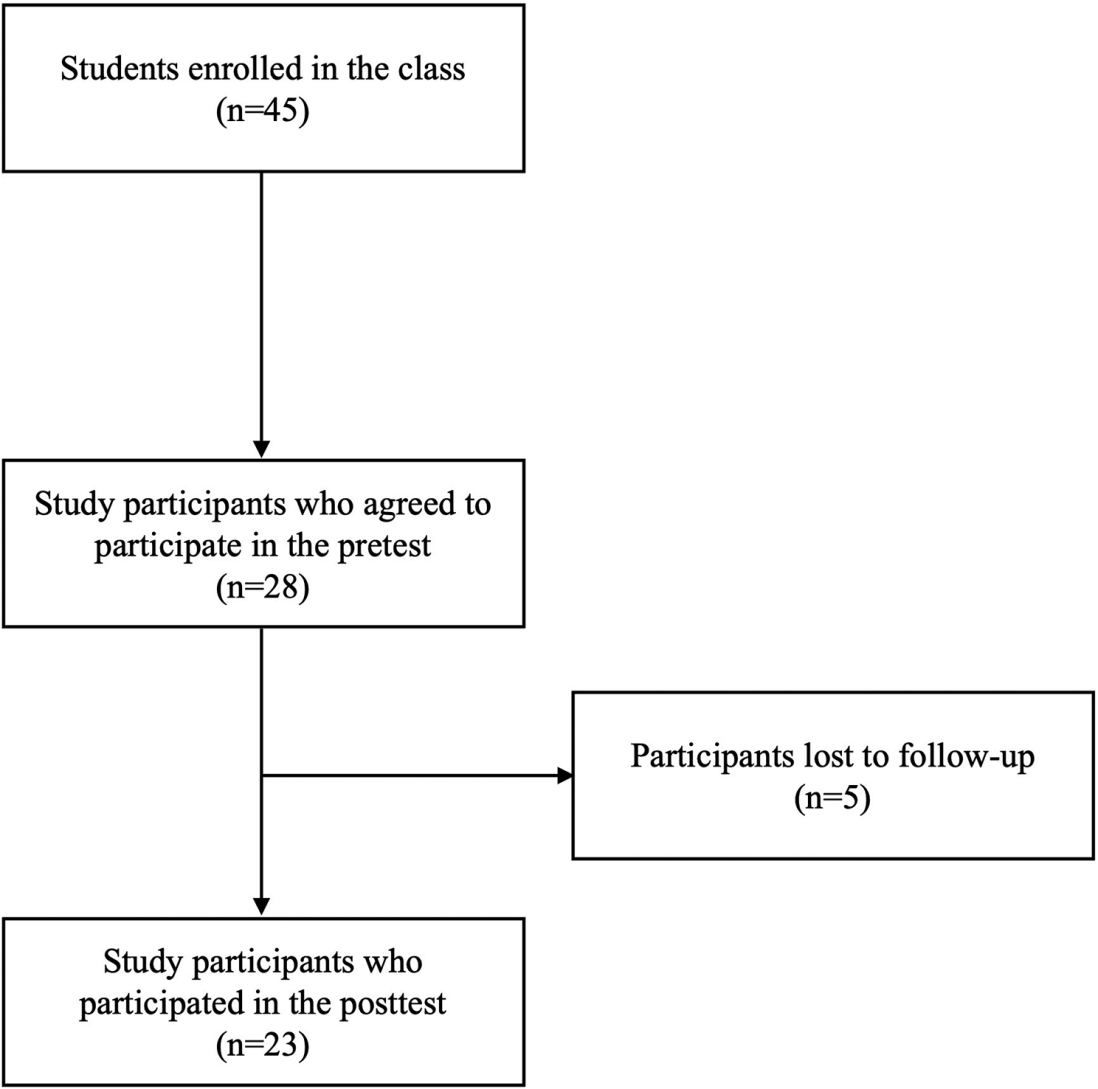
Participant Recruitment Flowchart

Out of the 45 enrolled in the course, 28 agreed to participate in this study. The initial survey (pretest) was completed by 28 students, and the follow-up survey (posttest) was completed by 23 students (Fig. 1). Consequently, the final analysis was conducted using the complete datasets from these 23 respondents. The sample size was calculated using G*Power 3.1.9.2 software before starting the study. With a large effect size (Cohen’s *d* = 0.8), a power of 0.95, and a significance level of 0.05 [22], the minimum required sample size was determined as 19. Therefore, the sample size of the study was sufficient.

The pretest was administered at the beginning of the semester in September 2023, and the posttest was conducted at the end of the semester in December 2023. A focus group interview was conducted with 5 students at the end of the semester in December 2023. This study was approved by the Institutional Review Board at the authors’ institution (IRB No. 2309/001–005).

### “Global Health and Nursing” course

The “Global Health and Nursing” course was developed by the corresponding author of this study (YH). It was an elective 15-week course for undergraduate nursing students taught in English. A collaborative project-based learning methodology was the main teaching method in this course. Although there were several mini-lectures on global health, the students mainly used the class time to work on their group projects. The development of the course, including the core content and topics for group projects, was based on literature on global health education [23, 24].

The group projects involved group assignments made by the course director at the beginning of the semester; each group comprised 5 students. The students were provided with a list of topics from which they could choose their project topic. The list included topics such as access to health care, nutritional health disparities, advanced directives, health of immigrants, environmental and health issues, reproductive health, communicable diseases and health, and literacy and health. They were able to either choose a topic from the list or create their own after discussing their proposed topic with the course director. Then, each group selected a specific global health issue that they wished to work on within their chosen topic. For example, if a group selected “environmental and health issues,” they could select a specific topic such as “Fukushima nuclear wastewater release in 2023 and its potential health impacts.”

The course director provided guidelines to assist students in the group project. The goals of the group project were to state the nature of the selected global health issue, identify its potential solutions, and determine nurses’ roles in the global health issue. The objectives of the course were to acquire knowledge about a variety of global health issues abroad, to assess global health problems, to identify potential solutions for global health issues, and to discuss nurses’ roles in the field of global health. The course director monitored each group’s progress weekly, discussing their current status during class and providing appropriate guidance and feedback on the group projects. At the end of the semester, each group was evaluated via group project papers and presentations. The course director assessed each project based on several key factors: whether the paper clearly outlined its research question, whether accurate and recent data were used to describe a global health problem, how potential solutions for the issues were identified, and how the nurses’ roles in addressing these issues were articulated.

The course director actively encouraged students to utilize generative artificial intelligence (AI) software, such as ChatGPT, as a pedagogical tool to augment learning outcomes. Specifically, the students were allowed to leverage this generative AI software for various educational purposes, including initiating discussions, generating ideas, and assisting with their English writing for the final paper. This innovative approach aimed to enhance student engagement and understanding by integrating cutting-edge technology into the learning process, thereby facilitating a more interactive and supportive educational environment.

The course director implemented a system for students to document and report their usage of AI resources. Each week, students filled out a form detailing their use of these resources, which they submitted at the end of the semester. The purpose of this system was to monitor and evaluate how students integrated AI tools into their learning process, ensuring that these technologies were used responsibly and effectively to enhance their educational outcomes.

Additionally, students were required to provide citations in their final group project paper, indicating the use of any resources that were not their original work. The course director employed plagiarism-checking software to review the final papers, deducting points for any plagiarism identified that lacked proper citations.

### Measures

#### Participant characteristics

Data on participant characteristics were collected. These characteristics included age, sex, academic year, grades, level of English communication skills, experience of visiting abroad, experience of contact with other cultures within the country, previous education related to global health, experience with participation in collaborative project-based learning, and satisfaction with the nursing major.

#### Global health competency

Global health competency was measured by the Global Health Competency Scale developed by the Global Health Education Consortium & Association of Faculties of Medicine of Canada to examine medical students’ global health competencies [25]. In this study, we used a Korean-translated version of the scale [26]. The scale has 30 items, which are measured on a 4-point Likert scale from 1 to 4. The possible scores range from 30 to 120, and a higher score indicates a greater level of competency in global health. The Cronbach’s alpha value for the Korean version of the scale is 0.95 [26], and in this study, the Cronbach’s alpha value was 0.97.

#### Communication, problem-solving, and self-directed learning skills

Communication, problem-solving, and self-directed learning skills were measured by the Life Skills Scale developed by Lee and colleagues in 2003 [27]. The original scale has 3 dimensions: communication skills (49 items), problem-solving skills (45 items), and self-directed learning skills (40 items). When developed, each dimension showed good reliability [27]. The alpha values of communication, problem-solving, and self-directed learning skills were 0.80, 0.94, and 0.93, respectively. Lee and colleagues have encouraged the use of the individual dimensions separately in other research [27]. Therefore, each dimension was used as a separate variable. In this study, Cronbach’s alpha for communication skills was 0.87, for problem-solving skills was 0.89, and for self-directed learning skills was 0.94.

#### Focus group interview

A focus group interview was conducted after all grades were released at the end of the semester. The course director (YH) arranged and conducted the interview with the participants who wished to participate. The interview was conducted in one group consisting of 5 students. It was completed in Korean so that the students could express their experiences without any language difficulties. The interview was conducted via Zoom and lasted approximately one hour. Participants gave verbal consent for the audio recording of the interview session. A semi-structured interview was conducted to understand the nursing students’ experiences with collaborative project-based global health education.

Interview questions included the following: “How has the collaborative project-based global health education impacted your global health competencies?” “How has the collaborative project-based global health education affected your communication skills?” “How has the collaborative project-based global health education influenced your problem-solving abilities?” “How has the collaborative project-based global health education affected your self-directed learning skills?” “What do you think about the approach of collaborative project-based global health education?” “Did you use generative AI, such as ChatGPT, for the class? If so, what was the experience like?” During the interview, participants were encouraged to freely express any additional thoughts. When new ideas emerged, the interviewer employed follow-up questions to elicit further details.

### Data analysis

For quantitative analyses, the following methods were employed. First, descriptive analyses were conducted to describe the characteristics of the participants. Means, standard deviations, ranges, and percentages were used. Then, we assessed the normality of the scores for global health competency, communication skills, problem-solving skills, and self-directed learning skills. Paired *t*-tests were conducted to examine the effects of collaborative project-based learning on global health competency, communication skills, problem-solving skills, and self-directed learning skills. All statistical analyses were performed using SPSS^®^ software version 22.0. Statistical significance was set at a *p*-value less than 0.05.

To analyze the results of the focus group interview, qualitative content analysis was performed [28]. First, the interview was transcribed verbatim by one author (SL). Through a rigorous and iterative process of reviewing the original data, two authors of this study (SL and YH) identified initial codes. They then collaboratively worked to refine these codes into categories, from which subthemes and overarching themes were derived. This collaborative coding approach ensured methodological rigor and validity in analyzing the qualitative data. The analyses were conducted in Korean language, and the final themes and quotes were translated into English for this paper.

## Results

Table 1 describes the demographic characteristics of the participants. The mean age of the study participants was 22.64 ± 1.52 (Min–Max: 21–28). The majority of the participants were female (*n* = 25, 89.3%), sophomores (*n* = 17, 60.7%), and did not have any previous experience with global health (*n* = 18, 64.3%). Experience of visiting abroad or with other cultures within the country varied among the participants.

**Table 1:** Characteristics of the Study Participants (*N* = 28)

Characteristics	*n* (%) or M ± SD	Min–Max
Age	22.64 ± 1.52	21–28
Sex		
Male	3 (10.7)	
Female	25 (89.3)	
Academic year		
Sophomore	17 (60.7)	
Senior	11 (39.3)	
Grades^†^		
3.0 ≤ — < 3.5	11 (39.3)	
3.5 ≤ — < 4.0	14 (50.0)	
≥ 4.0	3 (10.7)	
Level of English communication skills		
Low	12 (42.9)	
Moderate	10 (35.7)	
High	6 (21.5)	
Experience of visiting abroad		
None	8 (28.6)	
1–2	12 (42.9)	
3–4	3 (10.7)	
≥ 5	5 (17.9)	
Experience of other cultures within the country		
No	9 (32.1)	
Yes	19 (67.9)	
Previous education related to global health		
No	18 (64.3)	
Yes	10 (35.7)	
Experience of participation in collaborative project-based learning		
No	4 (14.3)	
1–2 times	9 (32.1)	
3–4 times	10 (35.7)	
≥ 5 times	5 (17.9)	
Satisfaction with the major		
Low	4 (14.3)	
Moderate	9 (32.1)	
High	15 (53.5)	

^†^ Notes: 4.3 represents the highest achievable grade

**Fig. 2:**
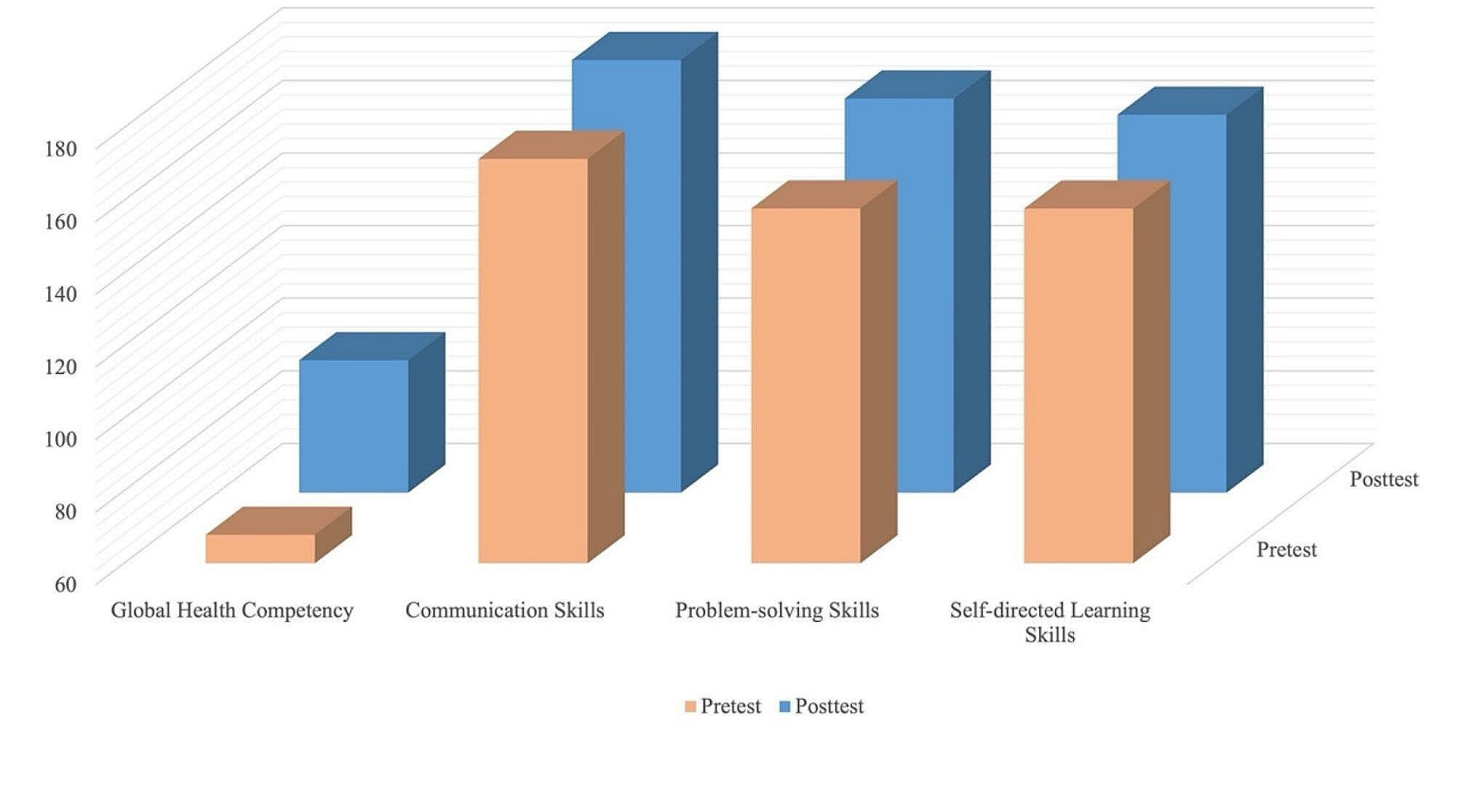
Comparison of scores in global health competency, communication, problem-solving, and self-directed learning skills (*N* = 23)

By evaluating the effectiveness of the course through paired *t*-tests, we found that the collaborative project-based learning approach produced statistically significant improvements in several key areas (Table 2; Fig. 2). There was a pronounced enhancement in global health competency (*t* = − 10.646, *df* = 22, *p* < 0.001), indicating a significant impact of the course on students’ understanding of global health issues. Moreover, communication skills improved significantly (*t* = − 2.649, *df* = 22, *p* = 0.015), suggesting that the course was effective in enhancing the ability of students to exchange ideas and cooperate with others. Another positive outcome was the improvement in problem-solving skills (*t* = − 3.453, *df* = 22, *p* = 0.002), reflecting a meaningful advance in the students’ capacity to tackle complex issues and think critically. Additionally, self-directed learning skills improved (*t* = − 2.375, *df* = 22, *p* = 0.027), underscoring the effect of the course in fostering independent learning among the students.

**Table 2:** Effectiveness of the Curriculum (*N* = 23)^†^

	Pretest	Posttest	Min–Max	t	df	*p*
Global Health Competency	67.87 ± 13.00	96.52 ± 10.70	Min–Max: 30–120	−10.646	22	**0.000**
Communication Skills	171.43 ± 17.06	179.22 ± 19.29	Min–Max: 49–245	−2.649	22	**0.015**
Problem-Solving Skills	157.78 ± 16.56	168.61 ± 17.01	Min–Max: 45–225	−3.453	22	**0.002**
Self-Directed Learning Skills	157.74 ± 24.89	164.17 ± 24.48	Min–Max: 45–225	−2.375	22	**0.027**

^†^ Notes: Only those participants who provided information for both pretest and posttest were analyzed

As a result of analyzing the focus group interview, three overarching themes that describe the experience of the collaborative project-based learning approach were identified: Theme 1: Promoting global health competency, Theme 2: Fostering life skills through collaborative projects, and Theme 3: Recommendations for future classes (Table 3).

**Table 3:** Summary of Themes and Subthemes of the Study

Themes	Subthemes
Promoting global health competency	● In-depth exploration by participating in collaborative projects
	● Expanding nursing students’ perspectives
	● Recommendations for future classes
Fostering life skills through collaborative projects	● Process of reconciling perspectives
	● Enhancement of critical thinking skills
	● Active learning and engagement
Recommendations for future classes	● Use of generative AI software
	● Course director’s involvement

### Theme 1. Promoting global health competency

#### In-depth exploration by participating in collaborative projects

Students experienced a substantial deepening of their knowledge and understanding through the collaborative projects. The collaborative project-based learning approach challenged them to engage with complex and unfamiliar topics, diverging from the traditional teaching methods that they were accustomed to. The traditional Korean teaching method in nursing includes one-way lectures from the lecturer to students. This shift required them to delve deeply and focus narrowly on specific issues, which they found to be a difficult yet rewarding process. By navigating the collaborative projects throughout the semester, the students felt that their global health competencies were significantly enhanced. The opportunity to analyze and think critically about global health issues provided a valuable learning experience, contributing positively to their educational growth and the development of their global health capabilities.

*“When selecting a topic for the group project, we encountered topics that were unfamiliar, and selecting a topic was challenging. However, this experience led us to study in a very focused and in-depth manner, which I think had a positive impact on enhancing our global health competencies.” (Student 4)*.

Participating in a collaborative project provided the students, who were accustomed to the traditional lecture-type teaching methods in nursing school, with a new learning experience.

### Expanding nursing students’ perspectives

The course facilitated a significant broadening of the nursing students’ views related to global health, allowing them to gain a deeper understanding and empathy toward situations around the world. Students expressed an appreciation for the exposure to a wide array of global health issues, which they had not previously encountered to such an extent. This exposure expanded their horizons beyond the local or national context and contributed to a more comprehensive grasp of health as a global entity.

*“The biggest takeaway for me was realizing that there are so many different health issues around the world, which has helped broaden my perspective significantly.” (Student 3)*.

Additionally, the students valued the integration of global health education into their curriculum, which is traditionally dominated by a clinical focus on direct patient care. In Korea, a diverse global health curriculum is lacking, and less than half of nursing schools offer an undergraduate curriculum dealing with global health issues [19]. By learning about global health issues in their elective course, they felt better prepared for the broader nursing context.

### Theme 2. Fostering life skills through collaborative projects

#### Process of reconciling perspectives

The students reported that communication was challenging as they were required to collaborate on the projects. However, they also expressed that their communication skills improved after they finished the project. For example, when there was a diversity of interests within the group, it led to extensive group discussions and the reconciliation of diverse perspectives.

*“Each team member had a lot of topics they were interested in…We communicated a lot with each other. I felt that my communication skills improved during that process.” (Student 1)*.

One student reported that she learned the importance of explicit communication. The student recognized the need for more nuanced communication skills, especially regarding integrating and refining the collective inputs into the final product.

*“The most difficult part was communicating during the final paper revisions. It was hard to grasp exactly what direction the feedback from each person was aiming for, and it was also a vague issue how to convey this to others…” (Student 4)*.

Therefore, the process of reconciling perspectives was an iterative learning experience that underscored the importance of clear and effective communication in achieving a unified vision in collaborative work. Participating in collaborative projects provided the students, who were accustomed to traditional lecture-type teaching methods, with opportunities to hone their communication skills.

### Enhancement of critical thinking skills

The collaborative projects underscored a significant growth in student abilities to engage deeply with complex issues facing the global community. By addressing real-world problems as a group, students experienced an increase in their awareness of global challenges and honed their critical thinking capabilities.

*“It wasn’t just about searching for a topic and finding all the related materials. It involved first identifying countries related to the topic, then focusing on the issues that have arisen in those countries, and the policies being implemented to solve those problems. Narrowing down the scope in this way to find and interpret data was greatly helpful in enhancing my research and analytical skills.” (Student 3)*.

The collaborative environment fostered by the team projects thus played a crucial role in developing students’ critical thinking skills, enabling them to approach global health challenges with an analytical and solution-oriented mindset.

### Active learning and engagement

The students had a transformative educational experience, from conventional lecture-based learning toward a dynamic and participatory approach. The students shared their journey of engaging deeply with the course material. One student noted the unexpected depth of understanding gained through reading papers, researching materials, and preparing presentations, in contrast to merely studying from textbooks for exams. Another student emphasized the novelty of their experience in conducting thorough research, including finding and analyzing multiple papers on the same subject, which led to a broader exploration of related topics.

*“Initially, I thought I wouldn’t study much for this subject since there were no exams. Contrary to my expectations, engaging in activities like reading papers, researching materials, and preparing presentations offered a depth of understanding far beyond what textbook study for exams could provide.” (Student 1)*.

### Theme 3. Recommendations for future classes

#### Use of generative AI software

Students reported diverse experiences with generative AI software. These experiences ranged from highly beneficial to eliciting skepticism, illustrating the diverse ways in which students interacted with and perceived the value of generative AI in their studies.

One student leveraged ChatGPT for initial guidance, particularly for outlining and collecting related materials for their part of the project. This approach suggests that generative AI can serve as a starting point for research, offering a foundation to build upon and modify for specific academic needs. Another student found ChatGPT extremely useful for exploring cases, precedents, and solutions related to their project topic and appreciated the varied answers and insights it provided. This active engagement with the tool highlights its potential as a versatile resource for collecting information and generating ideas.

*“The most significant aspect of using ChatGPT lies in its ability to yield diverse responses based on the type of question posed. For instance, when inquiring about strategies to address a sensitive topic like do-not-resuscitate (DNR) orders in Korea, ChatGPT can provide extensive insights. It may suggest examining policies from other countries, offering a comprehensive foundation for considering the implementation of analogous strategies within the Korean context.” (Student 2)*.

Furthermore, a student highlighted that the use of ChatGPT was limited to English translation tasks within their group, and another student expressed their distrust of ChatGPT, citing its unreliability as a major concern, which led them to avoid using the tool altogether.

Overall, the landscape of student interactions with ChatGPT was nuanced, ranging from enriching research and idea generation to cautious or limited use. This reflects the evolving role of AI tools in educational settings and their varying impact on student learning and project development. Such diversity in use and opinion underscores the need for ongoing exploration into the efficacy and appropriateness of AI software use in future coursework.

### Course director’s involvement

There was a range of student perspectives on the desired level of instructor involvement in the learning process. Some students appreciated the current level of involvement, which was hands-off, allowing them the autonomy to establish their own detailed timelines. This independence in managing their projects was perceived as beneficial, offering them the opportunity to develop self-regulation and project management skills.

Other students expressed a preference for more active intervention from the course director, such as the provision of a clear, structured timeline to guide them through the projects during the semester. This guidance was seen as a way to ensure consistent progress and help manage their workload effectively. Additionally, some students advocated for more assertive and direct feedback on their projects, including critical assessments. These students valued detailed and candid input, even negative, believing that such engagement could drive improvements and enhance the quality of their work.

*“I believe that our learning capabilities could be enhanced if we received more feedback or were posed with sharper questions that challenge us. Personally, I’m open to and see the benefit of more assertive feedback.” (Student 2)*.

## Discussion

This study was conducted to evaluate the effectiveness of global health education that utilized a collaborative project-based learning approach. This study found that the collaborative project-based learning approach was effective, especially in improving students’ skill sets.

### Effect of the collaborative project-based learning approach

The results of this study confirm the effectiveness of the project-based learning approach that was reported in previous literature [9, 29]. Many positive outcomes of applying a project-based learning approach have been reported [9, 29]. One study reported that project-based learning was more effective in improving academic achievements than traditional teaching methods [29]. As project-based learning is inquiry-based and fosters student autonomy, it allows students to discover problems and find solutions on their own [8, 30], which leads to improvements in motivation [31] and competency [32]. However, these results are mainly from other professional fields, and little knowledge is available on how project-based learning is effective specifically for nursing students. This study filled this gap in the literature by showing that project-based learning is an effective approach in the field of nursing as well. Furthermore, considering that project-based learning prepares students for real-world challenges [33], this method could significantly advance the professional development of nursing students by equipping them with practical skills for real-world settings. However, as the school environment, community support, or access to resources can influence project-based learning, nurse educators should consider providing a supportive environment and ensuring equal accessibility for such resources.

In addition to the project-based learning approach, the course integrated a collaborative approach so that students worked with their peers on a project. Collaboration and partnering are key aspects of global health competencies, as global health requires a diverse range of health professionals to work together [15]. Previous studies showed that collaborative learning has many positive effects, such as improving academic performance and enhancing student engagement [34–36]. Moreover, it can enhance cooperative skills among students [31], and effective communication, which is a critical skill for nurses [37, 38]. Previous studies that applied collaborative learning to nursing students’ clinical practicum confirmed its effectiveness in enhancing nursing competencies [39, 40]. Therefore, nurse educators who instruct courses on global health should consider applying a collaborative learning approach to improve communication skills among nursing students.

In this study, the collaborative project-based learning approach also improved problem-solving skills. This result is in line with previous studies, including systematic reviews and meta-analyses, reporting that problem-based learning improves overall thinking skills among nursing students [41–43]. Providing students with opportunities to contemplate real-world problems and potential solutions can increase their critical thinking skills. Therefore, nurse educators should consider exposing students to real-world problems to increase the students’ problem-solving skills.

### Recommendations for the future

Several recommendations emerged from the focus group interview. First, there was a range of student experiences with the use of generative AI software, such as ChatGPT. Student experiences varied significantly. While some found it extremely helpful, others expressed skepticism about its reliability. The interview revealed diverse usage patterns of generative AI among the students. For instance, some utilized it for in-depth exploration, employing detailed and sequential questioning techniques.

Educational technologies have been widely used in nursing education including problem-based learning [30, 44]. Generative AI software can be an effective supplementary learning tool in certain situations, but exploring whether it is being used effectively in project-based learning in nursing is necessary. Generative AI can be a learning partner when integrated into project-based learning and transform a competition-focused education system into a collaborative one [45]. Students can successfully perform a given task, gain confidence, and increase their participation through immediate feedback from AI learning partners, which can promote nursing education when appropriately used [46]. Further research is needed to determine whether generative AI can enhance learning outcomes for students. Specifically, these studies should explore how these technologies can be effectively integrated into curricula to maximize their potential benefits and address the unique challenges of global health training.

Another consideration for future classes includes the extent of the instructor’s involvement. Although the instructor’s support is essential in project-based learning, student choice and autonomy should be valued as well [8]. As mixed opinions were expressed by the students about the course director’s involvement in the collaborative projects, course instructors should consider the degree of their involvement when applying a project-based learning approach. Based on the findings, we recommend maintaining students’ autonomy while setting deadlines throughout the course so that students can keep it as a minimal guideline.

Moreover, exploring the cost-effectiveness of implementing collaborative project-based learning approaches in nursing education remains an essential area for future research. While this approach seems promising in enhancing student competencies, the financial and resource implications of widespread adoption in diverse educational settings warrant thorough investigation. For example, studies that analyze the scalability of such approaches and their impact on faculty workload and institutional resources could provide valuable insights into their practical viability. Additionally, challenges, such as managing group dynamics and workload distribution within collaborative settings, must be carefully considered. These factors can significantly influence the efficacy and sustainability of project-based learning methodologies.

Additionally, the long-term impact of collaborative project-based learning approaches on preparing nurses for global health careers should be further investigated. Understanding the immediate educational outcomes and how these approaches equip future healthcare professionals to handle complex global health challenges over the course of their careers is crucial.

#### Strengths and limitations

This study makes several contributions. First, although there is a lack of information available on applying a collaborative project-based learning approach in global health education, this study showed the effectiveness of this approach. Nursing instructors should consider adopting this teaching approach for global health education at the undergraduate level. Second, because this study employed a multi-method of quantitative and qualitative approaches, the effectiveness of the course was comprehensively assessed.

The study has several limitations as well. First, a pilot trial was not conducted before the study. Including a pilot trial could have enhanced the credibility and validity of our approach. Second, because the design of the study was a one-group pretest–posttest design, it did not include a control group for comparison, which limits the ability to attribute the observed changes directly to the intervention without considering other external factors. In future studies, randomized controlled trials should be considered to examine the approach’s effectiveness. Third, the study was conducted over a single semester; therefore, this 15-week course might not have been sufficient to observe long-term changes in student skills and knowledge. Future studies should consider extending the intervention duration to an academic year to evaluate its effect. Additionally, the long-term impact of the course was not observed in this study, which could have been possible by including time series results. Future studies should employ a longitudinal design so that the prolonged effects of the collaborative project-based learning approach can be assessed. Including follow-up assessments at multiple intervals post-intervention will enable better understanding of the sustainability of learning outcomes. Furthermore, integrating qualitative methods such as interviews or focus groups could provide deeper insights into how and why these approaches impact student learning over time. These strategies will not only enhance the robustness of the findings but also contribute to a more nuanced understanding of the educational interventions’ effectiveness.

Another limitation of this study was that it relied on the students’ self-reported data to examine the impact of collaborative project-based learning. Self-reported data can be susceptible to bias, and the improvements observed in global health competency, communication skills, problem-solving skills, and self-directed learning skills may reflect students’ perceptions rather than actual enhancements. Therefore, the findings must be interpreted with caution, as the reported improvements might not fully capture the students’ real skill development. Future studies should include objective measures in addition to self-reported data. Lastly, this study was conducted with a small sample of undergraduate students in Korea, and caution should be applied when interpreting the findings and considering their generalizability. Future studies with a larger and more diverse sample of nursing students are needed, and it is essential to test the approach in various educational settings to enhance generalizability and applicability.

### Implications

This study highlights the effectiveness of collaborative project-based learning in significantly enhancing critical competencies in global health among nursing students. Notably, the competencies enhanced include an in-depth understanding of global health issues, improved communication skills such as idea exchange and teamwork, as well as elevated problem-solving and self-directed learning abilities. These improvements demonstrate the potential for more engaging and effective educational practices. However, implementing such approaches comes with challenges, including the need for adequate resources, instructor training, and adaptation to varying learning environments and student backgrounds. While this study demonstrated positive short-term outcomes, the long-term impacts of these competencies on professional practice remain to be explored. Future studies should, therefore, include longitudinal follow-up assessments to evaluate the sustainability of the learned skills over time. Additionally, given the diversity in educational settings, replicating this study across different contexts is crucial to verify its effectiveness and adaptability. We recommend that educational policymakers and curriculum developers consider these factors when integrating collaborative project-based learning strategies within nursing education programs. Furthermore, randomized controlled trials should be conducted to rigorously evaluate the approach’s effectiveness and facilitate its wider adoption.

## Conclusions

The collaborative project-based learning approach was effective in global health education, especially in increasing students’ global health competencies and improving communication, problem-solving, and self-directed learning skills. Although they found it challenging, students were satisfied with the collaborative project-based learning approach, as it allowed them to delve deeply into learning global health issues. Therefore, nurse educators should consider applying this methodology to global health education for undergraduate nursing students. Future research employing a randomized controlled trial is warranted to further determine the effectiveness of the approach.

## Data Availability

The datasets generated and analyzed during the current study are not publicly available due ethical restrictions but are available from the corresponding author on reasonable request.

## Abbreviations

AI: artificial intelligence
